# Tuft cells promote human intestinal epithelium regeneration as reserve stem cells after irradiation

**DOI:** 10.1186/s13619-024-00214-w

**Published:** 2024-12-09

**Authors:** Yehua Li, Mengxian Zhang, Xianrun Ma, Ye-Guang Chen

**Affiliations:** 1 Institute of Biomedical Innovation, School of Basic Medical Sciences, Jiangxi Medical College, Nanchang University, Nanchang, 330031 China; 2 The State Key Laboratory of Membrane Biology, Tsinghua-Peking Center for Life Sciences, School of Life Sciences, Tsinghua University, Beijing, 100084 China

## Abstract

Intestinal epithelium regeneration is crucial for homeostatic maintenance of the intestinal functions. A recent study published in *Nature* uncovers tuft cells as an unexpected key player in the regenerative process. Human tuft cells, traditionally recognized for their involvement in immune defense and pathogen protection, were found to exhibit stem cell-like properties following radiation-induced injury. These cells not only resist damage but also have the ability to generate functional stem cells, promoting the repair of the intestinal epithelium. This finding suggests that tuft cells may function as a reserve pool of stem cells, essential for efficient intestinal regeneration after injury.

## Main text

The intestinal epithelium exhibits remarkable regenerative capacity to repair injuries, with Lgr5^+^ intestinal stem cells playing a central role in this process. Several cell types have been identified as contributors to this regeneration (Liu and Chen 2020). For example, the dedifferentiation of enterocytes (Tetteh et al. 2016) and secretory (Buczacki et al. 2013; Murata et al. 2020; van Es et al. 2012; Yan et al. 2017) progenitors has been shown to restore normal numbers of Lgr5^+^ stem cells after injury. Additionally, a subpopulation of quiescent Lgr5^+^ stem cells, marked by Mex3a, can reestablish stem cell function (Barriga et al. 2017). Furthermore, “revival” stem cells, characterized by transient expression of clusterin (Clu) and Sca1, have also been identified as a player in intestinal regeneration (Nusse et al. 2018; Yui et al. 2018).

Recently, Hans Clevers’ group at the University Medical Center Utrecht published a groundbreaking paper (Huang et al. 2024). The study sheds new light on the regenerative potential of the human intestinal epithelium, revealing that tuft cells, thought to play roles in immune defense and pathogen protection, possess remarkable stem cell-like properties after injury. The study demonstrates that tuft cells can resist radiation-induced damage and dedifferentiate into stem cells, facilitating the repair of the intestinal epithelium. Therefore, tuft cells may act as a “reserve stem cell reservoir” for regenerating damaged intestines.

To generate a model for visualizing and tracking the dynamics and functions of human intestinal tuft cells, Clevers and colleagues analyzed previously published single-cell RNA sequencing (scRNA-seq) data and identified AVIL, encoding the actin-binding protein advillin, as a specific human tuft cell marker. Green fluorescent protein (mClover) was then fused to the C-terminal of AVIL to create a reporter organoid line. Using these reporter cells, they observed that type 2 immune cytokines IL-4/IL-13 stimulate existing AVIL^+^ cells to divide twice within 36 h, with a continued increase of KI67^+^AVIL^+^ cells over 7 days. The response to IL-4/IL-13 of tuft cells in human intestinal organoids is distinct from the increasing differentiation of Lgr5^+^ stem cells into tuft cells in the mouse intestine, highlighting the self-expanding nature of human tuft cells.

After selecting tuft cells based on the AVIL-Clover signal and performing scRNA-seq, the authors classified the tuft cells into four distinct states (tuft-1 to tuft-4) based on transcriptional profiling, with tuft-3 exhibiting high expression of proliferative genes such as KI67. Notably, IL-4/IL-13 signaling triggers the expansion of tuft cell states characterized by high proliferation (tuft-3) and immune regulation (tuft-4). The study also identified key transcription factors, including POU2F3 and TCF7, which are required for tuft cell development, and revealed that the transcription factor HMX2 is essential for tuft cell proliferation in response to IL-4 and IL-13.

The authors then discovered that purified single tuft cells from IL-4/IL-13-treated human intestinal organoids have the ability to form new organoids. Interestingly, these tuft cells rapidly downregulate tuft-specific genes (e.g., AVIL) and upregulated stem cell markers, such as Lgr5, when cultured in the expansion medium. The sorted single tuft cells can function as progenitor cells and retain the capacity to generate all major intestinal epithelial lineages, including absorptive enterocytes, goblet cells, and enteroendocrine cells.

The regenerative capacity of tuft cells was further confirmed in response to injury. When organoids were exposed to irradiation, organoids containing tuft cells exhibited better recovery compared to POU2F3 knockout organoids that lack tuft cells. Additionally, IL-4 and IL-13 treatment enhances recovery of organoids from irradiation. Using lineage tracing, they demonstrated that AVIL^+^ tuft cells can give rise to the entire organoid after irradiation, showing that tuft cells play a critical role in human intestinal reconstitution following injury.

Finally, the study also indicates that tuft cells may play a role in fetal intestinal development. Fetal human intestines exhibit an increased frequency of KIT^+^ cells, which express a tuft cell signature similar to that observed in adult and pediatric intestinal tuft cells. These fetal tuft-like cells also exhibit a strong organoid-forming potential, suggesting that tuft cells may be involved in intestinal epithelial expansion during development.

## Conclusions

**Fig. 1:**
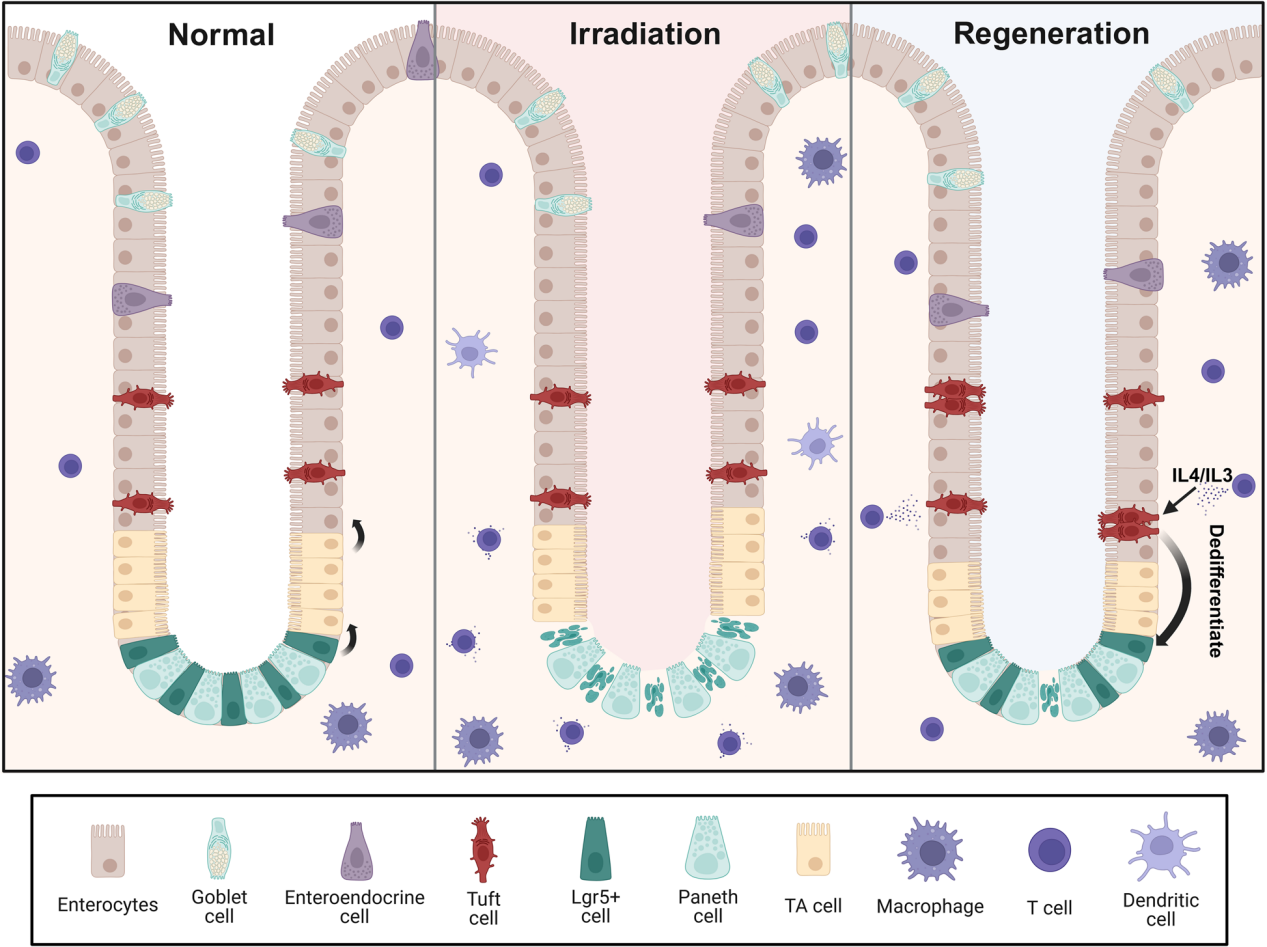
In normal conditions, Lgr5^+^ stem cells give rise to transient amplifying (TA) cells, which differentiate into all cell types within the intestinal epithelium. Lgr5^+^ stem cells are sensitive to irradiation-induced injury and can be lost following damage. After injury, the cytokines IL-4 and IL-13 can trigger tuft cells proliferation and dedifferentiation, regenerating Lgr5^+^ stem cells and mediating repair of the damaged intestinal epithelium. Created with BioRender

This study identifies AVIL as a specific marker for tuft cells, providing an important tool for future research on the role of these cells in both health and disease. The authors use a human-derived organoids, offering a great model for investigation of human intestinal biology, far better than previous animal-based models. In contrast to the traditional view that tuft cells are post-mitotic, this study shows that tuft cells can proliferate after injury, functioning as a reserve stem cell population in the human intestine and playing an important role in human gut regeneration following damage (Fig. 1). This study also highlights the potential of tuft cells as a novel source of regenerative stem cells for therapeutic applications. Additionally, the identification of cytokines and elucidation of a transcriptional control in the regeneration process open a new avenue for studying intestinal regeneration and treating related disorders, such as inflammatory bowel disease.

This study primarily utilizes human intestinal organoids, but it is unclear whether tuft cell-mediated intestinal regeneration occurs in vivo. An unanswered question is the source of IL4 and IL-13. Another intriguing aspect raised by this study is the role of the immune system in intestinal epithelial regeneration. Since IL-4 and IL-13, the cytokines released during type 2 immune responses, are key triggers of tuft cell expansion, it will be interesting to explore whether tuft cells-mediated intestinal epithelial regeneration is involved with type 2 immune responses. It is also worth examining whether in patients with inflammatory bowel disease, where the immune system is dysregulated, the epithelial regeneration suffers from abnormal interactions between immune cells and the intestinal epithelium.

## Data Availability

Not applicable.
